# Computational Discovery of Novel Monkeypox Virus DNA Polymerase Inhibitors from the Zinc20 Database

**DOI:** 10.3390/cimb48040347

**Published:** 2026-03-26

**Authors:** Ghaith H. Mansour, Belal Alshomali, Adam Mustapha, Diya Hasan, Maissa’ T. Shawagfeh, Laila Alsawalha, Wafaa Husni Odeh, O’la Ahmad Al-Fawares, Lara Al-Smadi, Muna M. Abbas, Mu’ad Al Zuabe, Mohd Effendy Abd Wahid

**Affiliations:** 1 Department of Allied Medical Sciences, Zarqa University College, Al-Balqa Applied University, P.O. Box 2000, Zarqa 13110, Jordan; 2 Department of Medical Applied, Amman Arab University, Amman 11953, Jordan; 3 Department of Microbiology, Faculty of Life Sciences, University of Maiduguri, Maiduguri 600104, Nigeria; 4 Department of Applied Biological Sciences, Faculty of Science, Al-Balqa Applied University, Al-Salt 19117, Jordan; 5 Department of Medical Laboratory Sciences, Faculty of Applied Medical Sciences, Irbid National University, Irbid 22110, Jordan; 6 Institute of Marine Biotechnology, Universiti Malaysia Terengganu, Kuala Terengganu 21030, Malaysia; 7 Faculty of Fisheries and Food Sciences, Universiti Malaysia Terengganu, Kuala Terengganu 21030, Malaysia

**Keywords:** monkeypox virus, molecular docking, molecular dynamics simulation, clinical trials, toxicity prediction

## Abstract

Monkeypox virus (MPXV) is emerging as a global public health concern due to its nature of spread. There are limited treatment options, as the sole drug for treatment is lacking, highlighting the need for new therapeutic options. The use of computer-aided drugs discovery such as molecular docking, molecular dynamic (MD) simulations and post-simulation analysis are important tools in identifying potential compounds that can target specific proteins of the virus, such as DNA polymerase to stop virus replication. This study employed molecular docking and molecular simulation with the aim to identify potential inhibitors for MPXV treatment from the ZINC Database. Molecular docking was performed using PyRx 0.8 version after virtual screening of the ZINC database using the Tranches tool; then, toxicity prediction of the selected compounds was performed using the ProTox-3.0 web server. Molecular dynamics simulation was conducted using GROMACS version 4.5 to evaluate the structural stability and dynamic behavior of the protein–ligand complex for the best interacting compound. Furthermore, post-simulation analysis was conducted using standard GROMACS utilities for visualizing time-dependent properties from MD simulations. A total of 16 compounds were shortlisted based on their molecular docking scores and interaction profiles with the monkeypox virus DNA polymerase (PDB ID: 8HG1). The leading compound, ZINC000019418450, demonstrated strong binding affinity (−7.4 kcal/mol). According to post-simulation analysis, all top compounds formed between one and five hydrogen bonds and up to eleven hydrophobic contacts with residues within the active site, thus providing strong geometric and energetic evidence for binding stability. Notably, our identification of ZINC000104288636 as a Class 6 compound with an LD_50_ of 23,000 mg/kg adds translational value by highlighting candidates with low predicted acute toxicity. Overall, this study lays a solid foundation for the rational design of next-generation monkeypox antiviral therapeutics. Future work is needed for experimental validation of prioritized compounds to assess their biochemical efficacy and pharmacological potential.

## 1. Introduction

Monkeypox virus (MPXV) is double-stranded DNA, a species of genus orthopoxvirus responsible for zoonotic infection, Mpox [1]. With its endemicity in western and central Africa, a global outbreak was reported in 2022 and 2024, where it was declared a public health emergency of international concern (PHEIC) by the World Health Organization [2]. It has continued to pose a public health threat due to exotic pet trade and international travel with person-to-person close contact as key transmission route [2]. The clinical presentation of MPXV involves symptoms such as fever, muscle pain, and rash-like blisters which tend to be severe in immunosuppressed individuals [3]. Typically, the symptoms are lesser but similar to smallpox, with the mortality rates ranging from 3 to 6% [4].

MPXV consist of a large genetic make-up encoding varying proteins responsible for the viral lifecycles and pathogenesis [5]. The replication machinery of the virus takes place within the host cytoplasm, beginning with the early expression of proteins such as transcription factors, DNA/RNA polymerases, capping enzymes, and methylases [3,5]. The virus employs DNA-dependent RNA polymerase (DdRp) pathway for replication, producing four distinct viral particles, intracellular mature virus (IMV), intracellular enveloped virus (IEV), cell-associated enveloped virus (CEV), and extracellular enveloped virus (EEV), responsible for infection and spread within human population [6]. Considering the essential role exhibited by MPXV DNA polymerase, inhibiting its activity would ultimately stop the DNA replication and spread of the virus [7].

While the sole drug for the treatment of mpox is still in its infancy, as a result of the emergency situation, drugs such as tecovirimat (TPOXX) targeting viral envelope proteins were approved. The key issue is that the replication machinery of MPXV is unknown, and questions of safety and efficacy of the drug on humans with mpox are not well established [3,8]. Fox et al. [9] systematically reviewed the ongoing efforts to assess the safety and efficacy of drugs for the treatment of mpox in both randomized control trials (RCTs) and non-randomized control trials for drugs such as tecovirimat, brincidofovir and cidofovir, NIOCH-14 and Vaccinia Immune Globulin Intravenous (VIGIV). The study did not identify any completed RCTs investigating the effectiveness of therapeutics for treating mpox, while on the other hand, very low-certainty evidence from non-randomized studies of small numbers of people indicates no serious safety signals emerging for the use of tecovirimat in people with mpox infection, but a possible safety signal for brincidofovir [9]. Therefore, there is need for continued search for candidate drugs while evaluating safety and efficacy.

The need for fast and cost-efficient drug development necessitates the deployment of computational and bioinformatics analyses for developing promising lead molecules. Advances in computational biology have revolutionized early-stage drug discovery, enabling large-scale screening of chemical libraries such ZINC Database which contain millions of compounds. This will hasten laboratory-based tests, and clinical trials can be used to further validate the identified drug candidates [10]. Computational methods such as virtual screening, molecular docking and molecular dynamics (MD) have been used to study many potential compounds for drug candidacy, which will give insight into the interaction between compounds, often known as receptors and ligands.

Studies have explored the use of computational studies to screen FDA-approved drugs (e.g., nilotinib, conivaptan) and phytochemicals (e.g., riboflavin, ellagic acid) against MPXV DNA polymerase [3,8,11,12]; many have relied primarily on docking score comparisons and descriptive MD stability assessments. In this study, we emphasize interaction mapping, residue-level flexibility analysis (Root Mean Square Fluctuation—RMSF), global structural deviation assessment (Root-Mean-Square Deviation—RMSD), and compactness evaluation (radius of gyration—RG) to interpret ligand binding behavior in structural terms. This integrated prioritization framework provides a mechanistically informed evaluation rather than a docking score-driven ranking.

## 2. Material and Methods

### 2.1. Compound Library Preparation

A virtual compound library comprising approximately 3.2 million small molecules was curated from the ZINC database (https://zinc.docking.org/tranches/home/# (accessed on 13 June 2025)), a free resource for commercially available compounds formatted for virtual screening [13]. The selection process was carried out using the “Tranches” tool on the ZINC20 platform with the following filtering criteria to ensure drug-likeness and docking suitability.

Molecular representations were limited to 3D conformations, which are appropriate for molecular docking protocols. Only compounds that were in-stock and commercially available were included, ensuring ease of procurement for downstream experimental validation. The reactivity filter was set to Standard, excluding highly reactive or unstable molecules. Physiological conditions were mimicked by selecting a reference mid-range pH and allowing compounds with formal charges ranging from −2 to +2 to reflect a realistic biological environment. Further filtering was performed based on physicochemical properties to comply with Lipinski’s Rule of Five and other common drug-likeness guidelines [14,15]. The LogP (partition coefficient) range was restricted to values between 0 and 3, and the molecular weight (MW) of compounds was constrained to be between 300 and 375 Daltons.

These parameters yielded a total of 3.2 million compounds. The selection grid was applied directly on the tranche matrix interface, and the resulting dataset was downloaded by exporting the tranche URLs using the platform’s download utility. The downloaded files, containing pre-processed 3D molecular structures in PDBQT format, were subsequently used for molecular docking analysis.

### 2.2. Molecular Docking Protocol

The docking of the curated ZINC compound library against the target protein was performed using PyRx version (version 0.8), an open-source virtual screening tool that integrates AutoDock Vina v.1.1.2 with a user-friendly interface as described by Agu et al. [16] and illustrated by Ayodele et al. [17].

### 2.3. Protein Preparation

The crystal structure of monkeypox virus DNA polymerase (PDB ID: 8HG1, resolution 2.80 Å), complexed with DNA polymerase processivity factor component A20, was obtained from the Protein Data Bank (PDB) [18]. To ensure optimal structural integrity, the 3D structure of the enzyme was prepared using UCSF chimera [19]. As described by Agu et al. [16], Ikuomola et al. [20] and Butt et al. [21], bound ligand was removed, and missing atoms, hydrogens, and loops were identified and corrected. A thorough structural assessment was conducted to verify the accuracy of all parameters, including atoms, residues, missing loops, and side chains. Incorrect chiralities were identified and corrected, and disulfide bonds and steric clashes were resolved. To further refine the structure, all water molecules (excluding those near the substrate binding site) and non-protein residues were removed. The prepared protein was then converted to PDBQT format for docking.

### 2.4. Ligand Preparation and Molecular Docking

The 3.2 million compounds, downloaded from the ZINC database in mol2 format, were imported into the PyRx project. Ligands were energy-minimized using Universal Force Field (UFF) with a conjugate gradients algorithm [22]. The docking grid box was centered on the protein’s active/binding site, as identified using CASTp (http://sts.bioe.uic.edu/castp/calculation.html (accessed on 13 August 2025)) binding pocket tools [23]. The grid box dimensions were set to adequately encompass the entire binding pocket, allowing for flexible ligand docking. The dimensions were 48 by 38 by 20 in Angstrom.

Molecular docking was performed using AutoDock Vina to predict ligand binding poses and estimate relative binding affinities within the MPXV DNA polymerase active site. The docking search space was defined with grid center coordinates at X = 120.7631 Å, Y = 156.3834 Å, Z = 135.9020 Å, and dimensions of 47.9942 Å × 38.0469 Å × 19.5572 Å to encompass the predicted catalytic region. The top-ranked binding pose based on predicted affinity was selected for interaction analysis. Sixteen (16) compounds with the lowest binding energies (i.e., highest affinities) were shortlisted. Binding interactions were further validated using LigPlot program (version 2.3), focusing on hydrogen bonding and hydrophobic interactions at the binding site. The LigPlot program automatically generates schematic 2-D representations of protein–ligand complexes from standard Protein Data Bank file input.

### 2.5. Molecular Dynamics Simulation and Post-Simulation Analysis

A 100 ns molecular dynamics (MD) simulation was conducted using GROMACS version 4.5 [24] to evaluate the structural stability and dynamic behavior of the protein–ligand complex for the best interacting compound (ZINC000019418450). MD simulation is a computational method that models the movement of atoms and molecules over time under defined physical conditions. The system was prepared using the CHARMM (Chemistry at HARvard Macromolecular Mechanics) force field [25], a widely used parameter set for biomolecular simulations that accurately models proteins, nucleic acids, and small molecules. The ligand topology was generated using CGenFF (CHARMM General Force Field) via CHARMM-GUI to ensure compatibility with CHARMM parameters.

The complex was solvated in a triclinic simulation box with TIP3P water molecules, a standard three-site water model compatible with CHARMM. The system was electrically neutralized by adding the required number of counterions (Na^+^ or Cl^−^). Energy minimization was conducted using the steepest descent algorithm to remove steric clashes and relax the system geometry. This was followed by equilibration under NVT (constant number of particles, volume, and temperature) and NPT (constant number of particles, pressure, and temperature) ensembles to stabilize the system at 300 K and 1 atm, using the V-rescale thermostat and the Parrinello–Rahman barostat, respectively.

A 100-nanosecond production MD run was then carried out with a 2-femtosecond time step, using the leap-frog integrator, which advances the positions and velocities of particles through time. Bond constraints were applied using the LINCS algorithm, and long-range electrostatic interactions were calculated using the Particle Mesh Ewald (PME) method. Simulation trajectory data were saved every 10 ps.

Post-simulation analysis was performed using standard GROMACS utilities. Root Mean Square Deviation (RMSD) was calculated to assess overall structural stability, Root Mean Square Fluctuation (RMSF) to evaluate residue flexibility, Radius of Gyration (Rg) to determine the compactness of the protein, and hydrogen bonding analysis to monitor stabilizing interactions between the compound and the protein. Output files in .xvg format were plotted using XMGRACE5.1.25, a graphical tool for visualizing time-dependent properties from MD simulations.

## 3. Results

### 3.1. Compound Selection Based on Docking Profile

Following virtual screening of approximately 3.2 million drug-like compounds from the ZINC database, a total of 16 compounds were shortlisted based on their molecular docking scores and interaction profiles with the monkeypox virus DNA polymerase (PDB ID: 8HG1). Docking simulations were conducted using AutoDock Vina implemented within the PyRx platform, with the protein treated as rigid and the ligands docked flexibly.

The selected top-ranked compounds exhibited binding affinities ranging from −7.4 to −7.1 kcal/mol. Given the relatively narrow energy window, compound prioritization was not based solely on docking scores but also on interaction patterns within the active site, including hydrogen bonding and hydrophobic contacts visualized through LigPlot analysis. This approach reduces overreliance on small differences in docking energy and strengthens structural interpretation.

In the broader context of small-molecule docking studies targeting viral polymerases, binding energies in the range of approximately −5 to −9 kcal/mol are commonly reported depending on ligand size and pocket characteristics [8,12,26]. According to Abduljalil and Elfiky [27], the average binding affinities of the nucleoside analogs against the active site of human monkeypox virus DNA-dependent RNA polymerase ranged from −5.92 to −6.59 kcal/mol. They presented Valopicitabine and Bemnifosbuvir as the best compounds, with average binding affinity values of −6.58 ±0.01 kcal/mol.

The values observed here therefore fall within the expected range for small-molecule–polymerase interactions. However, given the absence of structurally resolved, experimentally validated MPXV DNA polymerase inhibitors for direct benchmarking, the docking scores presented here are interpreted as preliminary indicators of binding compatibility rather than quantitative measures of inhibitory potency.

**Figure 1:**
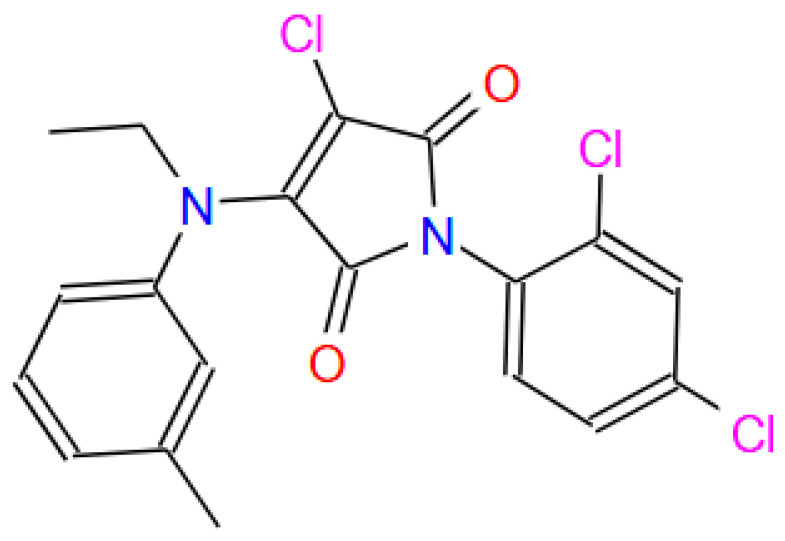
The 2D image top-performing compound, ZINC000019418450. SMILES: CCN(C1=C(Cl)C(=O)N(c2ccc(Cl)cc2Cl)C1=O)c1cccc(C)c1). Source: https://zinc.docking.org/substances/ZINC000001948450/ (accessed on 14 November 2025).

Table 1 summarizes the binding affinities and interaction characteristics of the top 16 compounds, as analyzed using the LigPlot software (version 2.3). The 2D image top-performing compound, ZINC000019418450, having Simplified Molecular Input Line Entry System (SMILES) CCN(C1=C(Cl)C(=O)N(c2ccc(Cl)cc2Cl)C1=O)c1cccc(C)c1), is presnted in Figure 1. It exhibited the lowest binding energy (−7.4 kcal/mol), forming four hydrogen bonds and seven hydrophobic interactions with the target protein. Other compounds demonstrated comparable binding energies, with multiple hydrogen bond and hydrophobic contacts, ranging between one and five hydrogen bonds and four and 11 hydrophobic interactions per ligand.

These ligands were selected based on their binding energy (kcal/mol) and their hydrogen bond (HB) and hydrophobic interaction (HI), as predicted by the LigPlot software (version 2.3).

### 3.2. Interaction Profile of Protein with the Selected Compounds

**Figure 2:**
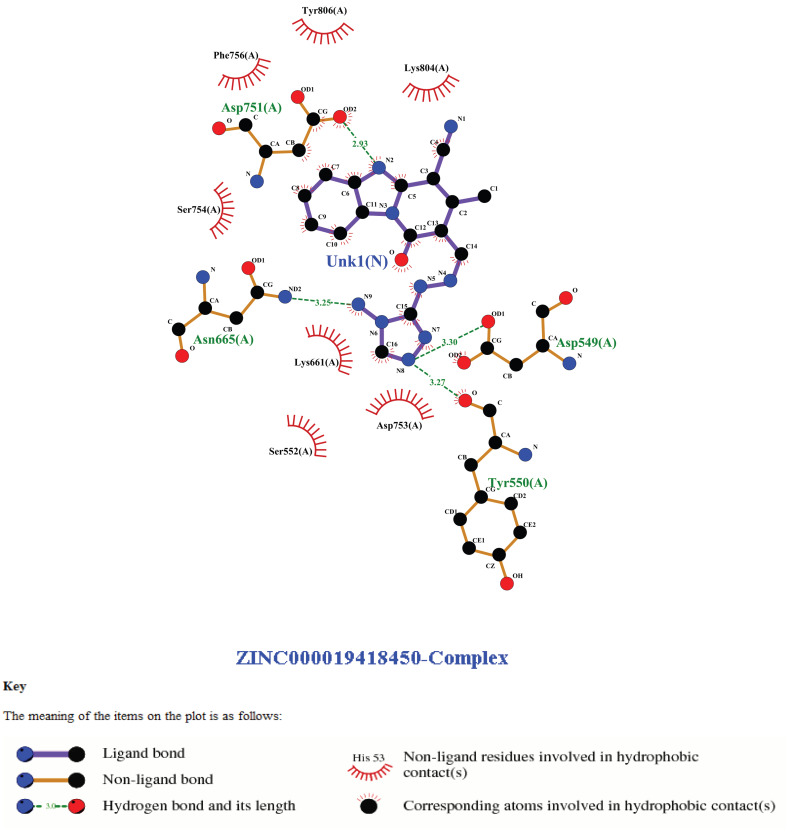
2D interaction diagrams of selected compounds with monkeypox DNA polymerase active site. LigPlot+ visualizations showing hydrogen bonding and hydrophobic interactions between top-ranked ligands and the catalytic pocket of monkeypox DNA polymerase (PDB ID: 8HG1). “Unk1(N)” denotes an ZINC00001948450 ligand labeled based on LigPlot+ annotation.

Interaction profiles of the 16 shortlisted ligands with monkeypox DNA polymerase were further examined using LigPlot, which generated schematic 2D representations of the binding modes. These visual analyses helped identify specific amino acid residues involved in hydrogen bonding and hydrophobic interactions at the active site of the enzyme. The LigPlot interaction profile of the first among the 16 shortlisted compounds is presented in Figure 2. The two-dimensional interaction diagrams of other shortlisted compounds are provided in Supplementary Materials, illustrating their binding modes and residue-level interaction patterns within the polymerase active site.

Key observations from the interaction profiling revealed that all compounds formed a variable number of non-covalent interactions, stabilizing their binding within the active pocket. Hydrogen bonds were frequently observed with residues located within or adjacent to the identified binding pocket, while hydrophobic interactions extended across several surrounding residues, contributing to enhanced ligand anchoring.

Although the detailed residue-level interactions are not listed in the current manuscript extract, the LigPlot visualizations were used to validate that all 16 compounds bound consistently within the predicted binding cavity. The quality and geometry of the binding interactions supported the inclusion of these ligands for further toxicity profiling and molecular dynamics simulation.

Comparative analysis of the shortlisted compounds (Supplementary Materials) reveals that while the core scaffolds differ in ring composition and substituent orientation, several shared structural features are apparent. Aromatic ring systems and polar functional groups capable of hydrogen bonding appear to contribute to active-site engagement. In particular, heterocyclic moieties and hydrogen bond donor/acceptor groups positioned toward key binding residues may represent promising fragments for further structure-based optimization.

### 3.3. Predicted Toxicity Profile of Selected Compounds

In silico toxicity prediction was performed to provide preliminary hazard assessment of the shortlisted compounds. The predicted LD_50_ values and corresponding toxicity classes should be interpreted as computational estimates derived from quantitative structure–activity relationship (QSAR)-based models rather than definitive indicators of in vivo safety. These predictions serve primarily as early-stage filters to identify compounds with potentially favorable safety profiles for further investigation.

The selected 16 compounds were subjected to in silico toxicity prediction using the ProTox-3.0 web server. This platform estimates potential toxicological classifications based on structural features and machine learning models, including LD_50_ values (lethal dose in mg/kg) and toxicity classes, ranging from Class 1 (most toxic) to Class 6 (least toxic) as presented in Table 2.

The toxicity classifications of the compounds ranged between Class 3 and Class 6, with the majority falling under Class 4, indicating a moderate level of toxicity. The compound ZINC000104288636 showed the least predicted toxicity, belonging to Class 6 with an LD_50_ value of 23,000 mg/kg. In contrast, ZINC000096196962 was the only compound classified under Class 3, with a relatively lower predicted LD_50_ of 298 mg/kg.

Other compounds such as ZINC000019418450, ZINC000022607266, and ZINC000012530038 showed LD_50_ values between 1100 and 1900 mg/kg, remaining within Class 4. The compound ZINC000020423493 was classified under Class 5, with a predicted LD_50_ of 3000 mg/kg, suggesting relatively lower acute toxicity.

These predictions provide an initial toxicological screening to guide further prioritization of lead compounds for experimental validation.

The toxicity profile of these selected compounds was predicted using ProTox-3.0 online server.

### 3.4. Molecular Dynamics Simulation of ZINC000019418450 with Monkeypox DNA Polymerase

Molecular dynamics simulation was performed to assess structural compatibility and conformational stability of the protein–ligand complex over time rather than to directly quantify binding free energy. The analyses presented (RMSD, RMSF, radius of gyration, and hydrogen bond monitoring) therefore provide insight into dynamic behavior and interaction persistence, but do not constitute direct thermodynamic measurements of binding strength.

To evaluate the dynamic behavior and structural stability of the top-ranked protein–ligand complex, a 100-nanosecond molecular dynamics (MD) simulation was conducted for the monkeypox virus DNA polymerase bound to ZINC000019418450. The simulation was performed using GROMACS version 4.5, employing the CHARMM force field for both protein and ligand components. The ligand topology was generated using CGenFF through the CHARMM-GUI server to ensure full compatibility with the force field parameters.

The protein–ligand complex was placed in a triclinic simulation box and solvated using TIP3P water molecules, a widely used three-point water model. To maintain electrical neutrality, appropriate Na^+^ or Cl^−^ counterions were added to the system. Prior to the production run, energy minimization was carried out using the steepest descent algorithm, eliminating steric clashes and optimizing the geometry of the system. This was followed by two phases of equilibration: first under NVT conditions (constant number of particles, volume, and temperature), and then under NPT conditions (constant number of particles, pressure, and temperature), using the V-rescale thermostat and Parrinello–Rahman barostat to maintain the system at 300 K and 1 atm, respectively.

The production MD run was performed for 100 nanoseconds using a 2 femtosecond timestep. Bond constraints were applied via the LINCS algorithm, and long-range electrostatic interactions were treated using the Particle Mesh Ewald (PME) method. System trajectory data were saved every 10 picoseconds for detailed post-simulation analysis.

**Figure 3:**
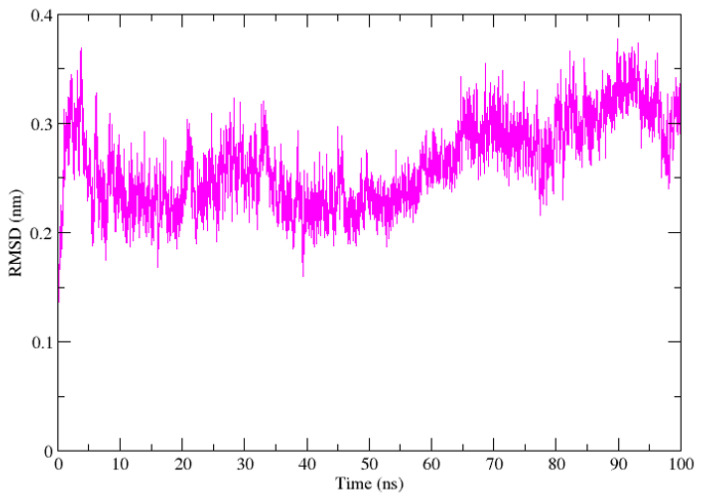
RMSD of ZINC000019418450–DNA polymerase complex Over 100 ns. Time-dependent plots showing backbone RMSD (nm) of the protein–ligand complex throughout the molecular dynamics simulation.

The RMSD of the protein backbone was monitored over 100 ns to assess global structural stability of the complex (Figure 3). Following an initial equilibration phase within the first ~10 ns, the RMSD stabilized and fluctuated within a range of approximately 0.20–0.35 nm, with an average value of ~0.26–0.30 nm during the production phase. No progressive drift was observed throughout the simulation, indicating preservation of the overall structural fold. The RMSD analysis showed moderate, indicating preservation of the overall protein fold without large-scale conformational disruption. The absence of large-scale deviations (>0.4 nm) suggests that ligand binding does not induce substantial conformational destabilization of the DNA polymerase structure.

**Figure 4:**
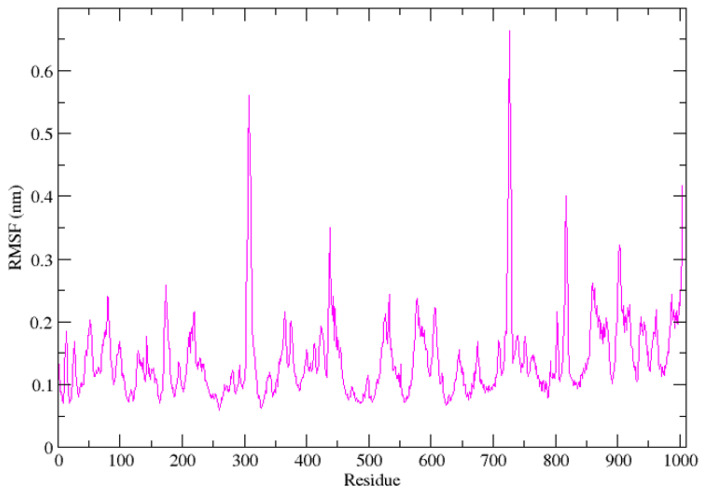
Residue fluctuation (RMSF) analysis of the complex. Line plots illustrate the RMSF profile of individual amino acid residues in the protein.

Also, the RMSF analysis was performed to evaluate residue-level flexibility across the simulation trajectory. The majority of the residues exhibited fluctuations below 0.20 nm, indicating relatively low backbone mobility. Two localized regions (Figure 4) demonstrated elevated flexibility: residues approximately 295–310 (peak ~0.55–0.57 nm) and residues approximately 715–730 (peak ~0.65–0.67 nm). These peaks correspond to flexible loop or surface-exposed segments rather than the structural core. Notably, residues outside these regions maintained fluctuations predominantly between 0.08 and 0.20 nm, suggesting preservation of the structural framework associated with catalytic and substrate-binding regions. The absence of widespread elevated fluctuations supports structural compatibility of ligand binding. The RMSF analysis (Figure 4) revealed that most residues exhibited low flexibility, with localized peaks corresponding to loop or surface-exposed regions rather than core catalytic residues This suggests that ligand binding does not destabilize the structural core of the polymerase.

**Figure 5:**
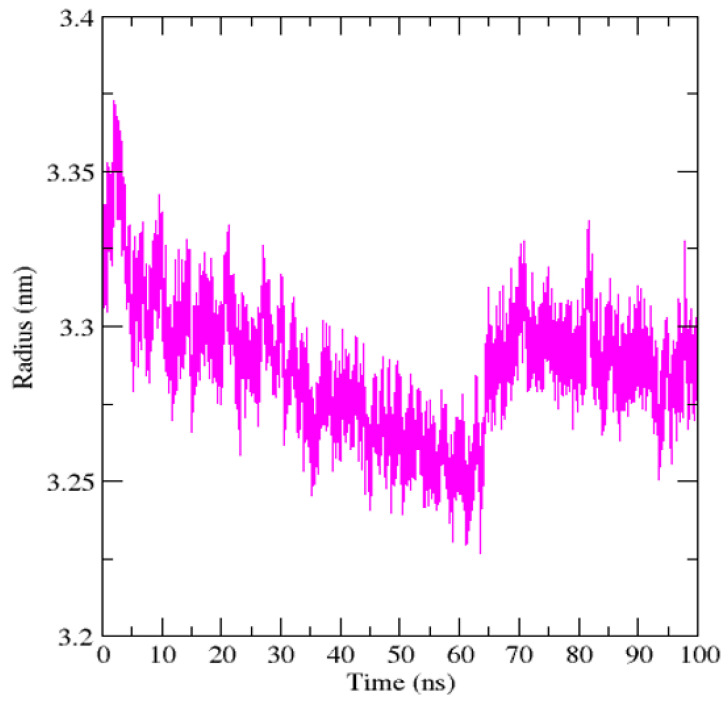
Radius of gyration analysis of ZINC000019418450–DNA polymerase complex over 100 ns. Time-dependent plots showing radius of gyration (Rg) of the protein–ligand complex throughout the molecular dynamics simulation.

The radius of gyration (Rg) was analyzed to evaluate the compactness of the protein–ligand complex. Throughout the 100 ns simulation, Rg values fluctuated within a narrow range of approximately 3.23–3.37 nm, with an average value around 3.28–3.30 nm. An initial slight decrease in Rg during the early phase was followed by stabilization, and no continuous expansion or collapse of the structure was observed. The relatively stable Rg profile indicates maintenance of global compactness and structural integrity during ligand association. The Rg remained stable throughout the simulation (Figure 5), indicating maintained compactness of the protein–ligand complex.

**Figure 6:**
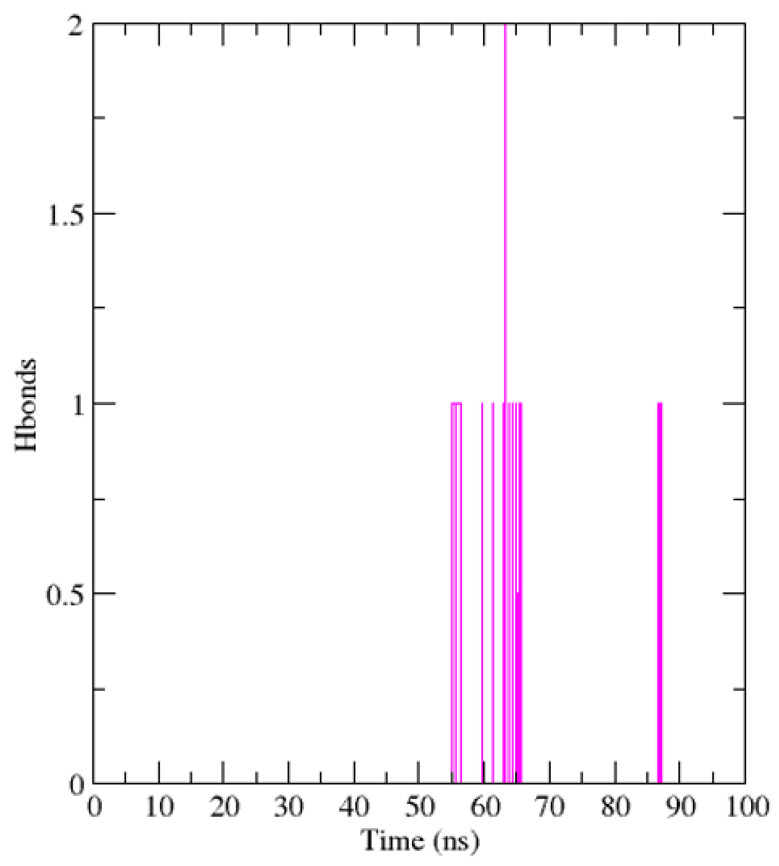
Hydrogen bond occupancy of the complex. Line plots illustrate the number of hydrogen bonds formed during the MD trajectory of individual amino acid residues in the protein.

Protein–ligand hydrogen bond interactions were monitored over the course of the simulation. The number of hydrogen bonds fluctuated between 0 and 2, with intermittent formation primarily observed between approximately 55–70 ns and occasional events near 85–90 ns. Persistent multi-hydrogen-bond networks were not observed (Figure 6). These results suggest that ligand stabilization is likely mediated by a combination of transient hydrogen bonding and nonpolar interactions rather than continuous hydrogen bond dominance. While hydrogen bond persistence was limited, the absence of abrupt dissociation events supports retention of ligand engagement within the binding pocket.

The results of these analyses were plotted using XMGRACE, enabling a detailed visualization of time-dependent structural behaviors within the protein–ligand complex.

## 4. Discussion

In the current study, a comprehensive computational framework was employed to identify potential inhibitors of monkeypox virus DNA polymerase through large-scale virtual screening, molecular docking, interaction profiling, toxicity prediction, and molecular dynamics simulation. The advancement of this work lies in integrating docking results with structural and dynamic interpretation to guide prioritization of MPXV DNA polymerase inhibitors. Rather than emphasizing screening scale or docking score magnitude, we focus on reproducible binding poses, interaction persistence, residue-level flexibility, and global structural stability. This approach contributes a more mechanistically informed framework for computational antiviral lead identification

The initial compound library, curated from approximately 3.2 million drug-like entries in the ZINC20 database, represents one of the largest screening efforts among comparable in silico antiviral or anticancer studies. For instance, Mathpal et al. [28] screened only 3180 FDA-approved ZINC compounds against SARS-CoV-2 main protease, while Onyango et al. [29] and Pal et al. [30] relied on ZINCPharmer to extract smaller pharmacophore-matching subsets from the ZINC15 database. In contrast, our workflow imposed rigorous pre-docking filtering criteria—adhering to Lipinski’s Rule of Five, filtering for charge, reactivity, and drug-like properties—to reduce redundancy and prioritize ligands with favorable ADME characteristics. This preliminary stringency aligns with the compound selection strategies employed by Taghvaei et al. [10], who screened natural compound analogs from ZINC for SENP1 inhibition, although their focus was narrower in chemical diversity and library scale.

The molecular docking phase implemented through AutoDock Vina within PyRx achieved binding affinities ranging from −7.4 to −7.1 kcal/mol among the top 16 ligands. These values compare favorably with those reported by Alanzi et al. [31], who observed similar energy ranges in their ROCK2 inhibitor screen, and Mathpal et al. [28], whose top FDA-repurposed hits against viral targets also fell within the −6.5 to −8.0 kcal/mol range. Our approach further incorporated CASTp-derived binding pocket prediction to enhance docking grid precision, a refinement absent in most reviewed studies. While Dantas et al. [32] and Hussein and Elkhair [33] performed docking studies, their reports did not document target site identification tools or provide evidence for binding pocket reliability, potentially weakening their structure-based assertions.

In addition to the docking comparisons, it is useful to consider known inhibitors of viral DNA polymerases as contextual benchmarks. Nucleoside analog antiviral agents such as cidofovir and brincidofovir, which have demonstrated activity against orthopoxviruses including monkeypox virus, target viral DNA polymerase during replication [34,35,36]. Previous computational and experimental studies evaluating inhibitors of viral DNA polymerases commonly report docking energies within the approximate range of −6 to −9 kcal/mol, depending on ligand size, binding pocket characteristics, and docking protocol [8,26]. Therefore, the binding energies obtained for the shortlisted ZINC compounds in the present study (−7.4 to −7.1 kcal/mol) fall within the expected range observed for small-molecule inhibitors of viral polymerases. While docking scores alone cannot confirm inhibitory potency, their consistency with previously reported antiviral polymerase interactions supports the structural plausibility of the identified compounds as candidate MPXV DNA polymerase inhibitors.

Notably, our study employed LigPlot v2.3 for detailed post-docking interaction profiling. Despite the prevalence of molecular docking across the literature, many authors only relied on general visualizations or energy-based metrics without detailed interaction maps, as could be seen in our LigPlot images for visualizing protein–ligand interactions. LigPlot analysis in our study, as similarly reported in the study of Rizvi et al. [37], revealed that all top compounds formed between one and five hydrogen bonds and up to eleven hydrophobic contacts with residues within the active site, thus providing strong geometric and energetic evidence for binding stability. This granular interaction profiling serves as a critical complement to docking energies, particularly when screening structurally diverse libraries.

The in silico toxicity profiling using ProTox-3.0 further refined the lead prioritization by categorizing compounds into toxicity Classes 3 to 6 and estimating LD_50_ values. The inclusion of this step aligns with recent efforts by Alanzi et al. [31] and Taghvaei et al. [10], to integrate early-stage ADMET assessments into virtual screening workflows. Notably, our identification of ZINC000104288636 as a Class 6 compound with an LD_50_ of 23,000 mg/kg adds translational value by highlighting candidates with low predicted acute toxicity. In contrast, several studies such as those by Hussein and Elkhair [33], Dantas et al. [32] and Putra et al. [38] omitted toxicity analysis entirely, thereby increasing the risk of downstream attrition.

Although computational toxicity prediction tools are valuable for early-stage screening, they are inherently limited by the training datasets and modeling assumptions used in their development. Predicted LD_50_ values do not account for complex biological factors such as metabolic activation, bioaccumulation, off-target interactions, species-specific differences, or long-term toxicity effects. Therefore, the toxicity classifications presented here should be regarded as preliminary indicators rather than confirmation of safety. Comprehensive in vitro and in vivo toxicological evaluation will be required to validate these predictions. In addition to toxicity considerations, pharmacokinetic properties such as absorption, distribution, metabolism, excretion (ADME), and potential drug–drug interactions may substantially influence therapeutic feasibility. Computational predictions cannot fully capture these complex biological processes. Accordingly, experimental pharmacokinetic profiling will be essential to determine clinical viability of the identified candidates.

The stability and conformational behavior of the top-ranked protein–ligand complex, ZINC000019418450 bound to DNA polymerase, were further evaluated through 100-nanosecond molecular dynamics simulation using GROMACS. RMSD analysis demonstrated maintenance of the overall protein fold throughout the simulation, indicating that ligand binding did not induce large-scale structural destabilization. RMSF analysis revealed localized flexibility primarily in loop or surface regions, while core structural elements remained comparatively stable. The radius of gyration remained consistent, supporting preservation of global compactness. Protein–ligand hydrogen bond analysis showed intermittent interactions over time, suggesting transient stabilizing contacts rather than persistent hydrogen-bond networks. Collectively, these results support structural plausibility of binding but do not, in isolation, establish quantitative binding affinity or inhibitory potency. These techniques are well-established in the field and mirror the simulation frameworks adopted by Mathpal et al. [39], Pal et al., [30] and Yousaf et al., [8] all of whom relied on 100 ns MD runs to evaluate dynamic stability and ligand retention. Our implementation of the CHARMM force field with CGenFF topology, TIP3P water model, and a dual-phase equilibration strategy further enhances simulation fidelity and replicates experimental thermodynamic conditions.

While several reviewed studies [10,39] extended their analyses with MM-PBSA free energy calculations, our study focused on trajectory-based stability metrics. Our MD simulations provide valuable insight into structural stability and interaction behavior, and the absence of binding free energy calculations such as MM-PBSA or MM-GBSA limits quantitative assessment of energetic favorability. Future work incorporating rigorous free energy estimation and experimental enzymatic assays will be necessary to validate inhibitory strength and confirm antiviral efficacy.

### Future Experimental Validation Plan

**Figure 7:**
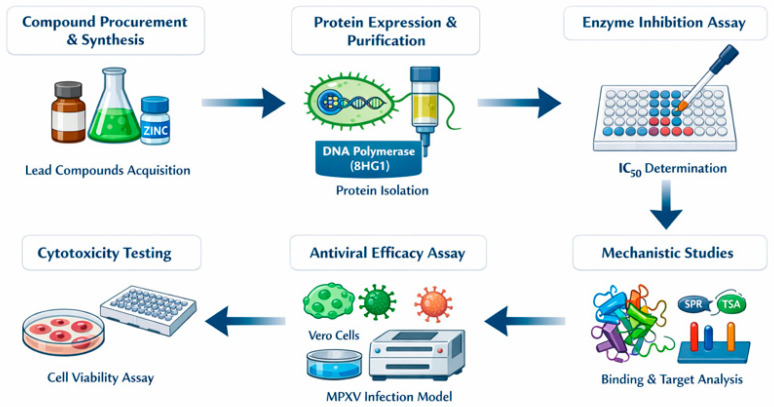
Experimental validation workflow.

To translate our in silico findings into tangible therapeutic leads, we propose a structured wet-laboratory experimental validation workflow targeting the top-ranked compound, ZINC000019418450, and a select few candidates from the top 16. This proposed validation scheme involves the following stages as summarized in Figure 7:

1. **Compound Procurement and Synthesis:** The prioritized compounds (ZINC000019418450, ZINC000104288636, and others) are readily available via commercial vendors as they were filtered from the ZINC20 “In Stock” subset. These compounds will be sourced or synthesized for further assays.
2. **Protein Expression and Purification:** The DNA polymerase (PDB ID: 8HG1) of MPXV, along with its processivity factor A20, will be cloned into a suitable bacterial expression system (e.g., *E. coli* BL21-DE3), followed by Ni-NTA affinity purification. Solubility tags such as MBP or GST may be used if solubility is an issue.
3. **In Vitro Enzyme Inhibition Assays:** The biochemical activity of the MPXV DNA polymerase will be assessed via a DNA polymerization assay using a fluorescently labeled primer-template system. IC_50_ values of each compound will be determined by measuring the inhibition of polymerase activity across a range of compound concentrations.
4. **Cytotoxicity Evaluation in Human Cell Lines:** HEK293T or HaCaT cells will be used to perform MTT or resazurin viability assays to assess the cytotoxicity of the compounds, corroborating in silico LD_50_ values predicted via ProTox-3.0. This will inform the therapeutic window.
5. **Antiviral Efficacy in Cell-Based MPXV Infection Model:** The most promising compound(s) will be evaluated in an in vitro MPXV infection model using Vero cells. Viral replication will be quantified using qPCR, plaque assays, or immunofluorescence staining of viral proteins.
6. **Mechanistic Validation:** To confirm that the antiviral activity is due to direct inhibition of DNA polymerase, pull-down assays, thermal shift assays (TSA), or surface plasmon resonance (SPR) can be employed to demonstrate direct binding between the compound and the viral DNA polymerase.

This integrative pipeline bridges computational predictions with empirical validation, ensuring translational relevance of our findings. The outcomes of these experiments will determine the compound’s potential as a lead candidate for further preclinical development.

## 5. Conclusions

In this study, a comprehensive in silico pipeline was employed to identify and evaluate potential inhibitors of monkeypox virus DNA polymerase, integrating large-scale virtual screening, molecular docking, interaction profiling, toxicity assessment, and molecular dynamics simulations. From an initial library of over 3.2 million drug-like compounds sourced from the ZINC20 database, 16 top candidates were shortlisted based on binding affinity and interaction stability within the active site of the target enzyme.

The leading compound, ZINC000019418450, demonstrated strong binding affinity (−7.4 kcal/mol), favorable hydrogen bonding and hydrophobic interactions as revealed by LigPlot analysis, low predicted toxicity (Class 4), and robust dynamic stability over a 100-nanosecond simulation. The consistency of these findings, coupled with the use of structure-guided docking and CASTp-defined binding sites, reinforces the credibility of the lead compounds for further investigation. The identified compounds represent structurally compatible candidates for further computational refinement and experimental validation as potential MPXV DNA polymerase inhibitors.

Future work will involve experimental validation of prioritized compounds to assess their biochemical efficacy and pharmacological potential. Overall, this study lays a solid foundation for the rational design of next-generation monkeypox antiviral therapeutics.

## Figures and Tables

**Table 1:** Docking scores and toxicity profile of selected compounds.

S/N	Ligand	Binding Energy	HB (LigPlot)	HI (LigPlot)
1	ZINC000019418450	−7.4	4	7
2	ZINC000006161162	−7.2	5	4
3	ZINC000008300079	−7.2	2	9
4	ZINC000022607266	−7.2	2	5
5	ZINC000096226427	−7.2	1	8
6	ZINC000006382830	−7.1	3	6
7	ZINC000012530038	−7.1	2	9
8	ZINC000016430344	−7.1	2	11
9	ZINC000020423493	−7.1	1	8
10	ZINC000020932323	−7.1	4	9
11	ZINC000096196962	−7.1	1	7
12	ZINC000096238892	−7.1	-	10
13	ZINC000104288636	−7.1	1	6
14	ZINC000254459141	−7.1	3	6
15	ZINC000408729544	−7.1	2	8
16	ZINC000585101594	−7.1	1	8

**Table 2:** Predicted toxicity classification and LD_50_ values of the top 16 screened compounds.

S/N	Compound	Predicted Toxicity Class	Predicted LD50
1	ZINC000019418450	4	1900 mg/kg
2	ZINC000006161162	4	418 mg/kg
3	ZINC000008300079	4	310 mg/kg
4	ZINC000022607266	4	1190 mg/kg
5	ZINC000096226427	4	800 mg/kg
6	ZINC000006382830	4	500 mg/kg
7	ZINC000012530038	4	1500 mg/kg
8	ZINC000016430344	4	400 mg/kg
9	ZINC000020423493	5	3000 mg/kg
10	ZINC000020932323	4	500 mg/kg
11	ZINC000096196962	3	298 mg/kg
12	ZINC000096238892	4	1100 mg/kg
13	ZINC000104288636	6	23,000 mg/kg
14	ZINC000254459141	4	1759 mg/kg
15	ZINC000408729544	4	750 mg/kg
16	ZINC000585101594	4	1350 mg/kg

## Data Availability

The original contributions presented in this study are included in the article/Supplementary Materials. Further inquiries can be directed to the corresponding author.

## Supplementary Materials

The following supporting information can be downloaded at: https://www.mdpi.com/article/10.3390/cimb48040347/s1.
