# Supplementary figures and images for: Computational Discovery of Novel Monkeypox Virus DNA Polymerase Inhibitors from the Zinc20 Database
Source: Curr Issues Mol Biol. 2026 Mar 26;48(4):347. doi: 10.3390/cimb48040347 (PMC13115257; doi:10.3390/cimb48040347)

**Plots**

ZINC00000616162-C  
ZINC000008300079-C  
ZINC000022607266-C  
ZINC000096226427-C  
ZINC000006382830-C  
ZINC000012530038-C  
ZINC000016430344-C  
ZINC000020423493-C  
**ZINC000020932323-C**

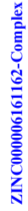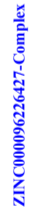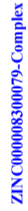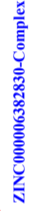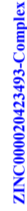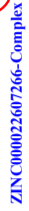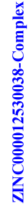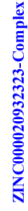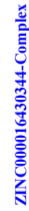

Supplement: Supplementary file 1 [file cimb-48-00347-s001.zip › supplementary of complex 1-9.pdf]

**Plots**

ZINC000096196962-C

ZINC000096238892-C

ZINC000104288636-C

ZINC000254459141-C

ZINC000408729544-C

**ZINC000585101594-C**

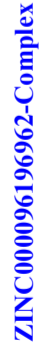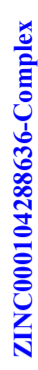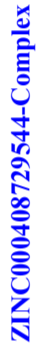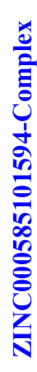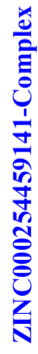

Supplement: Supplementary file 1 [file cimb-48-00347-s001.zip › supplementary of complex 10-15.pdf]
